# Targeted Inhibition of LPL/FABP4/CPT1 fatty acid metabolic axis can effectively prevent the progression of nonalcoholic steatohepatitis to liver cancer

**DOI:** 10.7150/ijbs.64714

**Published:** 2021-10-11

**Authors:** Haoran Yang, Qingmei Deng, Tun Ni, Yu liu, Li Lu, Haiming Dai, Hongzhi Wang, Wulin Yang

**Affiliations:** 1 Anhui Province Key Laboratory of Medical Physics and Technology, Institute of Health and Medical Technology, Hefei Institutes of Physical Science, Chinese Academy of Sciences, Hefei 230031, China.; 2 University of Science and Technology of China, Hefei 230026, China.; 3 Hefei Cancer Hospital, Chinese Academy of Sciences, Hefei 230031, China.; 4 Department of Anatomy, Shanxi Medical University, Taiyuan 030024, China.

**Keywords:** Nonalcoholic steatohepatitis, Hepatocellular carcinoma, Fatty acid metabolism, Gene differential expression, Metabolic reprogramming

## Abstract

**Rationale:** Nonalcoholic steatohepatitis (NASH), as one of the key stages in the development of nonalcoholic fatty liver disease (NAFLD), can directly progress to HCC, but the underlying mechanism is not fully understood.

**Methods:** Differentially expressed genes (DEGs) in each stage of disease development were studied through a GEO dataset deriving from a Stelic Animal Model (STAM), which can simulate the evolution of NAFLD/NASH to HCC in humans. GSVA analysis was performed to analyze the differentially expressed oncogenic signatures in each stage. A human NAFLD-related dataset from GEO database was utilized for gene expression verification and further validated in the protein level in STAM mice. Small molecule inhibitors were applied to STAM mice for investigating whether inhibition of the LPL/FABP4/CPT1 axis could prevent the occurrence of NASH-related HCC *in vivo*. Microsphere formation and clonal formation assays *in vitro* were applied to study if inhibition of the LPL/FABP4/CPT1 axis can reduce the viability of liver cancer stem cells (LCSCs).

**Results:** We found that upregulation of the LPL/FABP4/CPT1 molecular axis, as a fatty acid metabolic reprogramming process, occurred specifically during the NASH phase. GSVA analysis showed widespread activation of a large number of oncogenic signals, which may contribute to malignant transformation during NASH. Furthermore, inhibition of the LPL/FABP4/CPT1 axis could effectively delay the tumor growth in STAM mice. Cell assays revealed inhibitors targeting this axis can significantly reduce the sphere-forming, proliferation, and clonality of LCSCs.

**Conclusion:** These results suggest that activation of the LPL/FABP4/CPT1 axis is essential for LCSCs maintenance, which acts synergistically with a variety of up-regulated oncogenic signals that drive the hepatocyte-LCSCs transdifferentiation during NASH to HCC progression. Thus, targeting the LPL/FABP4/CPT1 axis may provide a potential direction for NASH-related HCC prevention.

## Introduction

Liver cancer is the second leading cause of cancer-related death among the most common malignancies worldwide 1, 2, which consists of heterogeneous groups of malignant tumors with different tissue characteristics and adverse prognoses 3. The main hepatocellular tumors include hepatocellular carcinoma (HCC), intrahepatic gallbladder tumor (iCCA), hepatoma, hepatocellular adenoma and pediatric tumor 4. It is estimated that the incidence of primary liver cancer ranges from 600,000 to 800,000 per year, accounting for 5.6% of all human cancers 5, among which HCC accounts for approximately 80%-90% of all primary liver cancers 2. The global pattern of HCC distribution reflects regional and ethnic differences, specific etiological factors, and socioeconomic status 6, 7. The incidence of HCC remains high and tends to increase globally 8. The basic mechanism of occurrence, development and metastasis of primary liver cancer remains largely unknown. While the incidence of HCC patients associated with hepatitis B virus (HBV) and hepatitis C virus (HCV) has gradually decreased due to the application and development of nucleotide-based therapies, the incidence of cryptogenic HCC continues to increase 9.

Increasing food intake and sedentary habits have significantly exacerbated the global epidemic of metabolic syndrome (MetS) 10, 11. It was observed that patients with MetS have a higher incidence of HCC than those without MetS 12. NAFLD, the result of liver involvement in MetS background, is becoming a part of it 13-15. HCC is the most neglected complication of NAFLD and perhaps the most clinically challenging complication. The increase of cryptogenic HCC is mainly related to NAFLD 16-18. NAFLD can develop from simple steatosis to NASH, then to liver fibrosis and even cirrhosis, and finally to HCC 19. However, most NASH progress to HCC without cirrhosis, suggesting that biological processes that promote tumorigenesis may occur during NASH.

Relevant studies have shown that genetic factors, diet and intestinal microorganisms, pro-inflammatory cytokines TNFα and IL6 are associated with the development of NAFLD/NASH to HCC 20. However, the exact mechanism has not been clarified and no effective drugs have been developed against these targets. NASH is regarded as a critical stage in NAFLD's transition to HCC, so revealing the molecular basis of NASH is crucial for effective disease prevention and treatment. Human specimens generally provide only a certain stage of the disease, and rarely provide information on the continuous progression of NASH to HCC. Using animal models for research overcomes this limitation. Although the high-fat, high-cholesterol (optional supplementation with sucrose and/or lipotoxic species) type diet is widely used to induce NASH in mice, it rarely induced the progression from NAFLD to HCC. The Stelic Animal Model (STAM) mice are the first animal model of NASH-related liver carcinogenesis resembling disease development in humans 21. It was found HCC developed in STAM mice is equivalent to stages B or C of the BCLC staging system in humans 22. From a clinical perspective, animal experiments that mimic the development of human HCC will facilitate the development of new therapies directly related to human liver cancer, thus providing more successful opportunities for the prevention and treatment of HCC. Analysis of DEGs at all stages of disease development in STAM mice will provide molecular information related to NASH-associated HCC. Besides, abnormal metabolism is a critical factor in the occurrence and development of tumors 23. Considering NAFLD is closely related to lipid metabolic disorder, the role of lipid metabolism abnormalities in NASH-related HCC remains largely unknown.

In this study, we conducted in-depth research on the progression of NASH to HCC by analyzing the DEGs at various stages in STAM mice liver tissues and HCC samples from The Cancer Genome Atlas (TCGA). We found a specific fatty acid metabolic reprogramming occurrence in the NASH stage. To investigate the importance of this metabolic reprogramming process, we systematically investigated the impact of molecular interventions targeting this metabolic pathway on tumorigenesis through *in vivo* and *in vitro* experiments, to offer potential solutions for the prevention of NASH-associated HCC.

## Results

### Identification of DEGs during NASH to HCC transformation by bioinformatics

**Figure 1:**
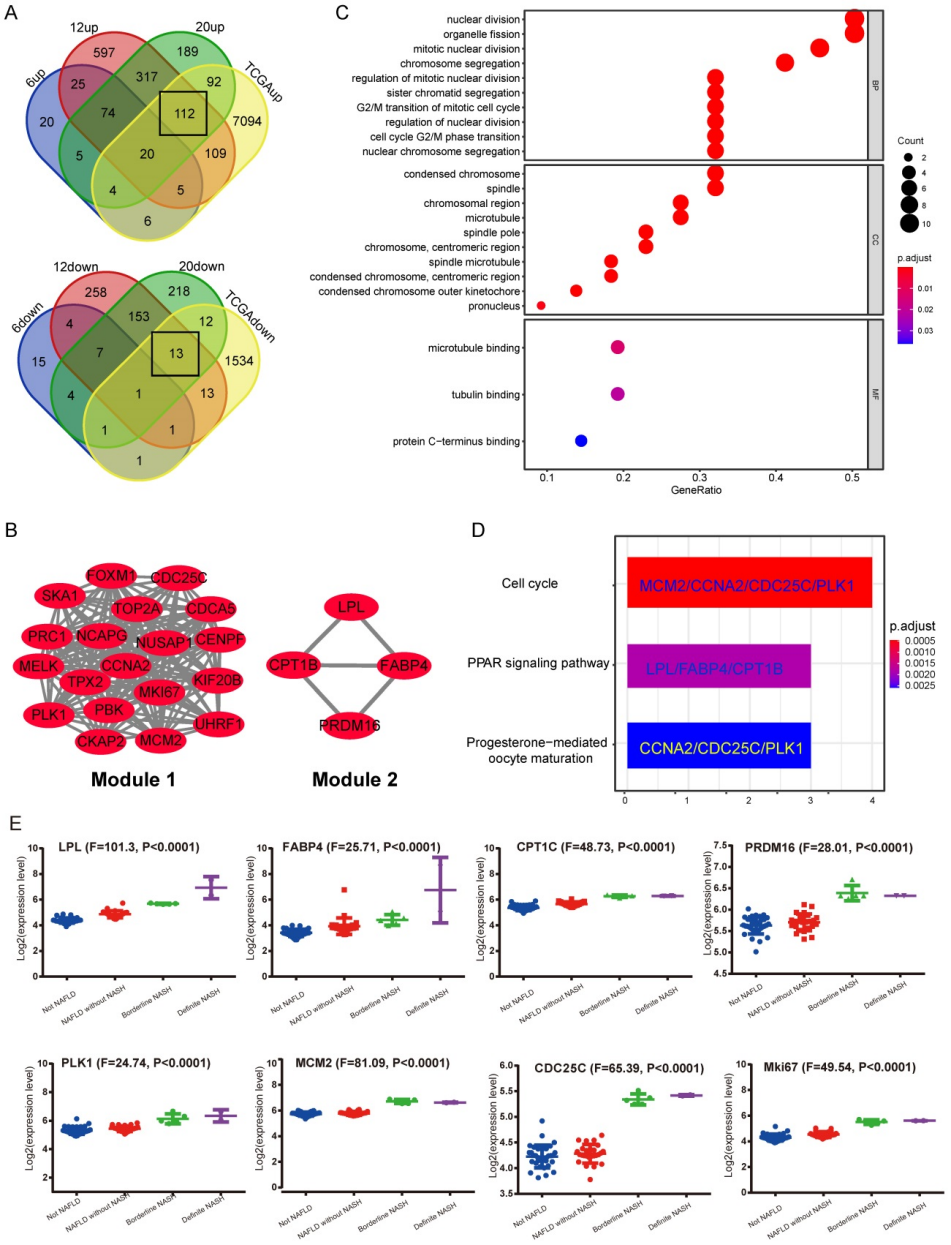
** Identification of shared differentially expressed genes (DEGs) in NASH and HCC. A,** Venn Diagrams were used to analyze common DEGs, including both upregulated and down-regulated, in liver samples of STAM mice (12W, 20W) and HCC samples from TCGA, but not altered in 6w-STAM mice. The number of common DEGs is shown in the black box.** B,** Top two functional modules (Module 1 and 2) were displayed after being analyzed by the MCODE plugin in Cytoscape software. **C,** GO analysis of genes contained in Module 1 and 2. **D,** KEGG analysis of genes contained in Module 1 and 2. Genes from Module 1 are mainly enriched in the process of “Cell cycle”, whereas genes from Module 2 are enriched in the “PPAR signaling pathway”. **E,** Expression levels of some common DEGs were verified in human liver samples, including normal (Not NAFLD), NAFLD without NASH, Borderline NASH, Defined NASH specimens (one-way ANOVA was used for statistical analysis).

In the GEO database, according to the study from Aline de Conti *et al* 24, the STAM mice dataset (GSE83596) contains gene expression information from NAFLD/NASH to HCC. The gene expression data of liver samples from STAM mice at 6, 12 and 20-week, corresponding to stages of simple steatosis, NASH with fibrosis and complete HCC, were extracted. Meanwhile, the HCC gene expression profile was derived from the TCGA database (LIHC-TCGA). The above data was put together for analysis using Venn Diagram. Considering that NASH is a critical pre-pathological stage, it is expected to contain key information that leads to the occurrence of NASH-related HCC. Therefore, we focused on DEGs that were cooperatively up-regulated or down-regulated in stages including NASH with hepatic fibrosis (12-week), complete HCC (20-week) and TCGA HCC data (LIHC-TCGA), but not altered in the simple steatosis stage (6-week) (Figure 1A). The results showed that most of the commonly shared DEGs were up-regulated. Kyoto Encyclopedia of Genes and Genomes (KEGG) pathway analysis indicated these up-regulated DEGs are most significantly enriched in the cell cycle pathway (Supplementary Figure S1A). Next, the protein-protein interaction (PPI) network was constructed by using the STRING tool (Supplementary Figure S1B). The core functional modules of the PPI network were then analyzed by the plug-in MCODE of the Cytoscape software. The two top functional modules are shown in Figure 1B. Module 1 is the functional proteome represented by MKi67 and PLK1 that regulates cell proliferation, and Module 2 is the functional cluster of proteins that regulate fat metabolism. Gene Ontology (GO) and KEGG pathway analysis showed that the genes containing in these two modules were just significantly associated with cell proliferation. The top enriched GO items are “nuclear division”, “condensed chromosome and microtubule binding” (Figure 1C). Enriched KEGG pathways are “Cell cycle”, “PPAR signaling pathway”, etc. (Figure 1D). These results strongly suggest that a significant change in NASH to HCC may be related to cellular processes regulating cell proliferation.

Subsequently, the results of the synergistic up-regulation of LPL/FABP4/ CPT1 molecules and some cell cycle-related genes were verified by liver biopsy samples of adolescents undergoing bariatric surgery in the GEO dataset (GSE66676). This dataset contains information on gene expression in liver tissues of human NAFLD/NASH patients at different stages, including cases of simple obesity (Not NAFLD, n=34), cases of NAFLD (NAFLD without NASH, n=26), marginal cases of NASH (Borderline NASH, n=5), and well-defined cases of NASH (Definite NASH n=2). The results indicated that with the progression of NAFLD, the LPL/FABP4/CPT1 molecules and some cell proliferative markers were generally upregulated since the NASH phase (Figure 1E). Lipoprotein Lipase (LPL) is a lipoprotein lipase that hydrolyzes very-low-density lipoprotein (VLDL) and triglyceride (TG) in chylomicron, releasing free fatty acid (FFA). Fatty Acid Binding Protein 4 (FABP4), which is a kind of fatty acid-binding protein that regulates intracellular FA transport and metabolism. CPT1 is a fundamental regulator of long-chain fatty acid β-oxidation, in which CPT1a, CPT1b and CPT1c are both members of the CPT1 family and have homology. Although CPT1c exhibits high sequence similarity to CPT1a and CPT1b, it is specifically expressed in neuron and cancer cell lines 25*.* Therefore, the putative role of CPT1c may confer cancer cells resistance to glucose- and oxygen-deprivation and serves as a promising therapeutic target. In this study, CPT1b was upregulated in mouse NASH, while CPT1c was upregulated in human NASH, indicating that the role of different CPT1 isoforms in tumorigenicity may be redundant. Furthermore, most cell cycle-related proteins in Module 1 have been widely reported to be associated with tumorigenesis. However, what role do LPL, FABP4 and CPT1 molecules enriched in the PPAR signaling pathway play in liver tumorigenesis? It is noteworthy that the progression of liver cancer depends on fatty acid metabolism and oxidative phosphorylation to promote the self-renewal ability and tumor-initiation properties of tumor-initiating stem cells (TICs) 26. Therefore, the synergistic upregulation of the LPL/FABP4/CPT1 molecules in the NASH stage may accelerate the occurrence of liver cancer by nourishing the TICs formation.

### The widespread activation of oncogenic signals during the NASH phase

**Figure 2:**
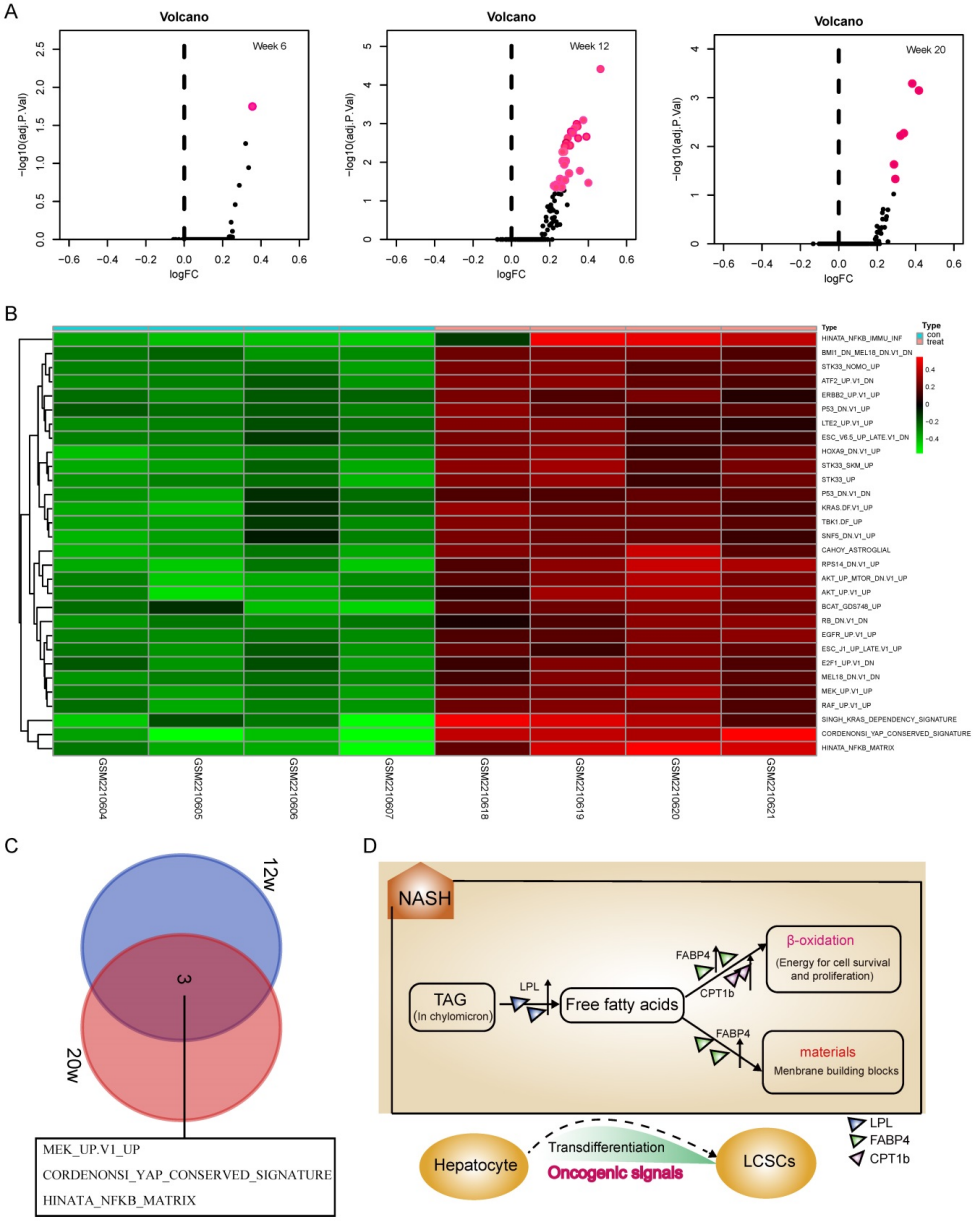
** GSVA analysis indicated a widespread activation of oncogenic signals during the NASH phase. A,** The volcanic map showed the changes of oncogenic signatures' expressions at each stage of the NAFLD/NASH-HCC development. **B,** Heatmap shows oncogenic signatures that changed at week 12. **C,** Venn diagram shows oncogenic signals that remained active at weeks 12 and 20. **D,** The hypothetical model: Activation of the LPL/FABP4/CPT1 metabolic axis is essential for NASH progression to HCC. During the NASH phase, multiple oncogenic signals are activated to drive the transdifferentiation of hepatocytes into liver cancer stem cells (LCSCs), while LPL/FABP4/CPT1 metabolic axis provides materials and energy to facilitate the proliferation and self-renewal of LCSCs.

Although activation of the LPL/FABP4/CPT1 metabolic axis may be beneficial for the maintenance of liver cancer stem cells (LCSCs), the genesis of TICs should involve more complex mechanisms that require the malignant transformation of normal hepatocytes. To understand what mechanisms prompt hepatocyte-LCSCs transdifferentiation, GSVA analysis was performed by comparing the expressions of oncogenic signatures between STAM and normal mice. The results indicated that a large number of oncogenic signatures were significantly upregulated during the NASH phase. On week 6, as shown in Figure 2A, there were almost no changes in the expression of oncogenic signatures, but on week 12, the liver tissue of STAM mice showed significant upregulations of a large number of oncogenic signatures (Figure 2A). As was shown in the heatmap (Figure 2B), there are 30 oncogenic signatures significantly upregulated at week 12, which involves various carcinogenesis-related pathways, including P53, EGFR, KRAS, ERBB2, YAP signaling, etc. Among these significantly enhanced oncogenic signatures, some may be directly related to cell transdifferentiation, such as the KRAS, YAP signaling. Importantly, it was noted that the three oncogenic signals remained upregulated until the late stage of tumorigenesis (week 20) (Figure 2C). They are MEK_UP.V1_UP, CORDENONSI_YAP_CONSERVED_SIGNATURE and HINATA_NFKB_MATRIX. These three oncogenic signals may play a more important role in liver carcinogenesis, not only in hepatocyte-LCSC transdifferentiation but also in tumor development. Collectively, the genesis of TICs may largely rely on the widespread activated oncogenic signals during NASH. As a synergistic process, the generated TICs require a material and energy supply to support their vitality, where activation of the LPL/FABP4/CPT1 signaling axis may play a role. Since abnormal lipid metabolism is essential for the activity of cancer stem cell maintenance 26, 27, we assumed that the activation of the LPL/FABP4/CPT1 metabolic axis may be an important promoter to facilitate the progression of NASH to HCC through collaboration with oncogenic signals. This metabolic reprogramming behavior may create a microenvironment conducive to the occurrence of HCC by providing material (fatty acids for building blocks) and energy (fatty acid oxidization), thus supporting the proliferating activity of tumor-initiating cells (Figure 2D).

### Verification of upregulation of LPL/FABP4/CPT1 at the protein level in STAM mouse liver

**Figure 3:**
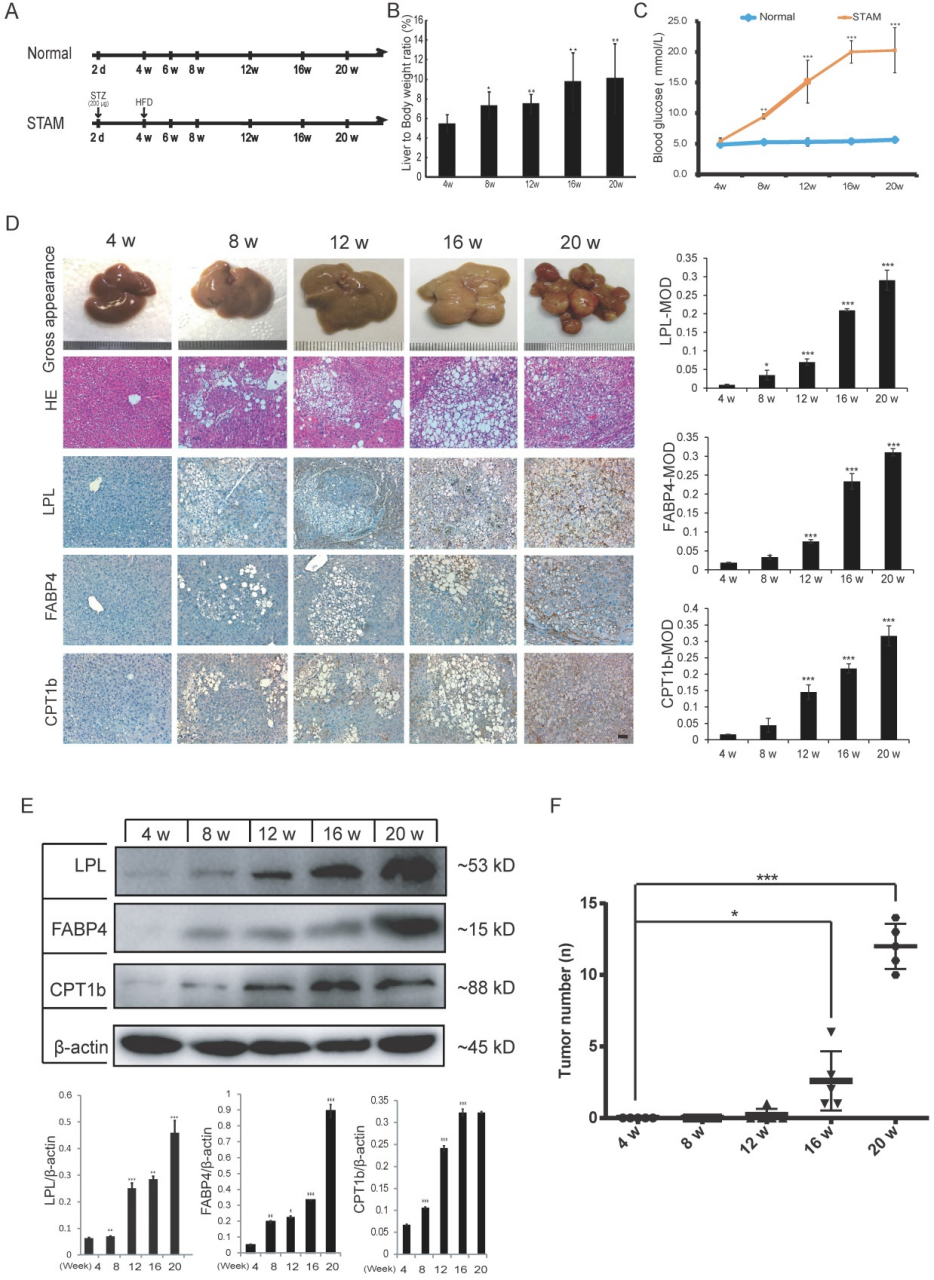
** The protein expression of LPL/FABP4/CPT1 during the progression of NAFLD was verified in the STAM mouse model. A,** Experimental schematic diagram for STAM mouse model establishment: In the Normal group and STAM group, the dose and time of administration are indicated by (⬇). **B,** In the STAM mice, the liver/body weight ratios at different stages (4w, 8w, 12w, 16w and 20w) were displayed. (*P < 0.05, **P < 0.01 vs 4w, n = 5 independent experiments). **C,** In the Normal group and STAM group, compared with 4w, the glycemic index of mice at different stages was analyzed (**P < 0.01, ***P < 0.001, n=5 independent experiments). **D,** Representative liver images of STAM mice at 4, 8, 12, 16 and 20 weeks. The panel includes gross appearance (scale bars, 1 mm), hematoxylin and eosin (HE) staining (scale bars, 100 μm), and immunohistochemistry (IHC) analysis with anti-LPL, FABP4, and CPT1b antibodies (scale bars, 100 μm). **E,** Western blot showed that the levels of LPL, FABP4 and CPT1b in the liver tissues of STAM mice increased gradually during NAFLD development, as showed at 4, 8, 12, 16 and 20 weeks. **F,** The number of liver tumors in the STAM mice during NAFLD development, as shown at 4, 8, 12, 16 and 20 weeks. Comparisons of data were performed with a paired two-tailed Student's t-test. Data are shown as mean ± SD. (*P < 0.05, **P < 0.01, ***P < 0.001 as compared with the value of the preceding point in time, n=3 in each group).

To further verify whether activation of fatty acid metabolism signal axis is an important step in NASH progression to HCC, we again used the STAM mice model for targeted inhibition experiments *in vivo*. STAM mice were constructed through streptozotocin (STZ) treatment and then fed high fat and glucose until 20 weeks. Meanwhile, the Normal group had a regular diet as a control (Figure 3A). Compared with the Normal group, the liver/body weight ratio and blood glucose level of STAM mice increased significantly (Figure 3B, C). Blood lipid-related index analysis showed that during the progression of NAFLD, the lipid correlation index of STAM mice gradually became disordered over time (Supplementary Figure S2A).

Liver tissues of STAM mice at 4, 8, 12, 16 and 20 weeks were examined based on hematoxylin-eosin (HE) and immunohistochemical (IHC) staining for LPL, FABP4 and CPT1 (Figure 3D). The pathomorphological analysis showed that the liver of STAM mice changed over time: At 4 weeks, the livers of STAM mice were reddish-brown, soft and smooth, similar to the Normal group. At 8 to 12 weeks, the livers of the STAM mice became larger, with a yellowish-brown appearance, white spots, and a slightly greasy cut surface. In mice aged 16-20 weeks, a single greater nodule or diffuse multiple nodules were present, involving most or even the entire liver. HE staining showed that the hepatocytes of mice at four weeks diverged outward from the central vein, the hepatic lobules were arranged densely and clearly, and there were occasionally bubbling fat droplets between the cells. However, a large number of vacuolar steatosis, swelling of liver cells were seen in the liver of STAM mice at 8 to 20 weeks.

IHC analysis combined with mean optical density (MOD) showed that the expression of LPL/FABP4/CPT1 molecules increased gradually during the period of NAFLD/NASH progression to HCC (Figure 3D). Western blot was further validated that the expression levels of LPL, FABP4 and CPT1, showed a gradually increasing trend during the progression period (Figure 3E). All three proteins are mainly expressed in the liver cancer cells (Supplementary Figure S3A). Specifically, LPL has mainly localized the periphery of cancer cells; CPT1 is mainly localized in the cytoplasm of cancer cells; FABP4 showed a certain degree of fuzzy staining in the cancer cells, which may be caused by its secretory status. FABP4 also exhibits some endothelial cell-like patterns in the tumor microenvironment, which is consistent with the previous study 28. Co-immunostaining of CPT1 with tumor stem cell marker EpCAM in the culture spheroids showed that they are co-localized in the LCSCs (Supplementary Figure S3A). The number of tumors in the liver tissue of STAM mice increased markedly from week 16 (Figure 3F). The above results demonstrated the STAM mice were successfully established to simulate the transformation from NASH to HCC. It also concluded that the LPL/FABP4/CPT1 molecules are up-regulated during the transition of NAFLD/NASH to HCC in the STAM mice at the protein level.

### Inhibitors of the LPL/FABP4/CPT1 molecules prevent the transformation of STAM mice to HCC

**Figure 4:**
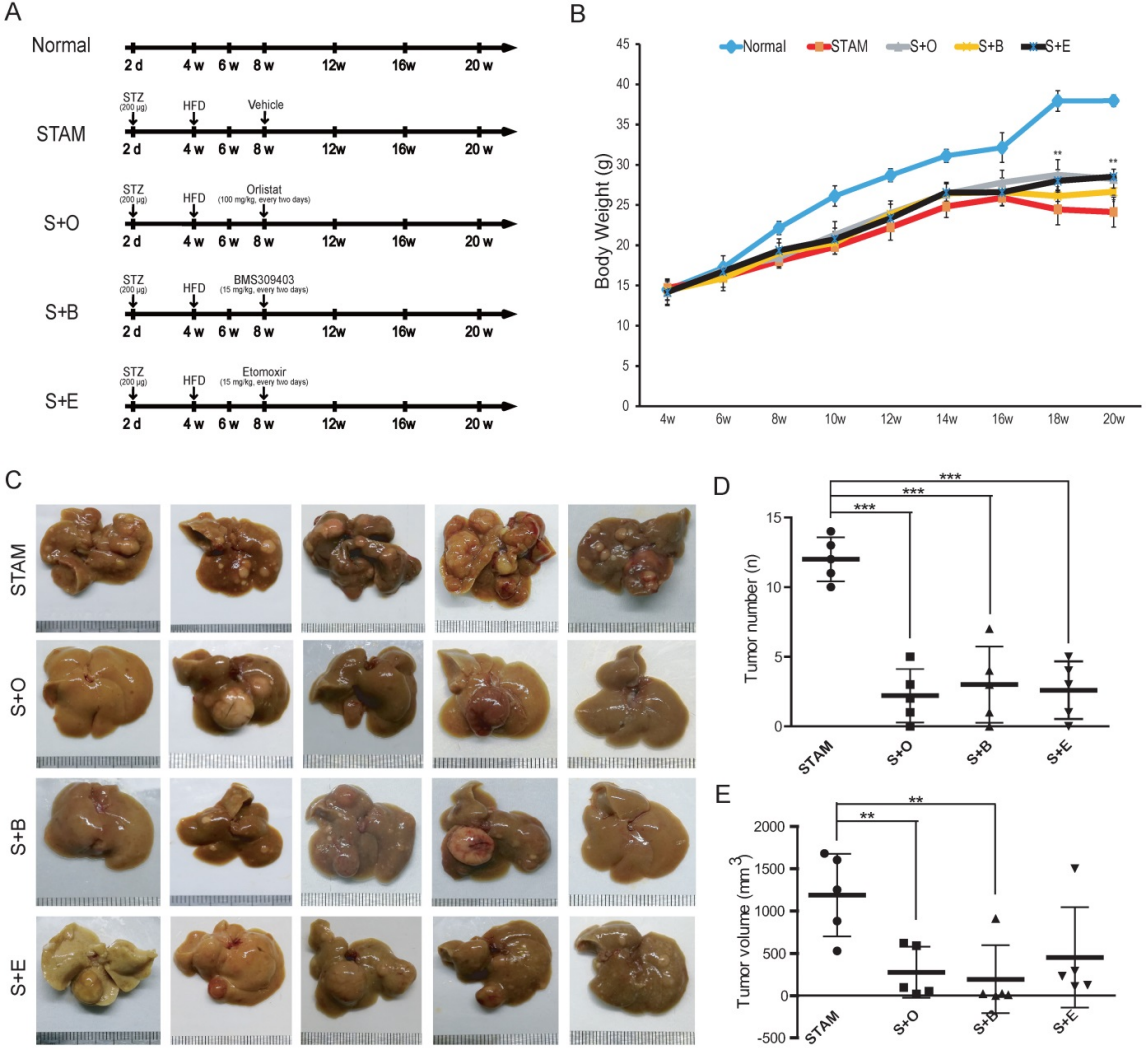
** Reduction of tumor burden in the liver of STAM mouse following inhibitors treatment. A,** Schematic diagram of the experimental process of normal group (Normal), STAM group (STAM), Orlistat inhibitor group (S+O), BMS4903403 inhibitor group (S+B) and Etomoxir inhibitor group (S+E). The doses and time points of drug administration are indicated by ⬇. **B,** Changes of body weight of mice in the experimental group (*P < 0.05, **P < 0.01 vs 4w, n=5 independent experiments). **C,** The gross appearance (Scale bars, 1 mm) of the liver in each group. **D & E,** The number and volume of liver tumor burdens in each group (a paired two-tailed Student's t-test was used for statistical analysis) (**P < 0.01, ***P < 0.001, n=5 in each group).

Next, we treated STAM mice with LPL/FABP4/CPT1 small-molecule inhibitors, including Orlistat, BMS309403 and Etomoxir, to determine whether the interference of this metabolic axis affects the occurrence of HCC. Three groups of STAM mice were set up for treatment with different inhibitors, including the LPL inhibitor Orlistat (S+O) group, the FABP4 inhibitor BMS309403 (S+B) group and the CPT1 inhibitor Etomoxir (S+E) group (Figure 4A). Compared to the mice in the normal diet group (Normal), the mice in the STAM group, S+O group, S+B group and S+E group all showed a trend of weight decline after 16 weeks. However, the statuses of mice in the S+O group, S+B group and S+E group were significantly better than those in the STAM group. Notably, mice body weight in the inhibitor-treated groups was significantly higher than that of the STAM group, especially after 16 weeks (Figure 4B). There was no significant difference in liver weight ratio and serum-related indexes between the STAM group and the other three inhibitor groups (Supplementary Figure S4A, B). Tumor burdens were ameliorated in three inhibitor groups compared to the STAM group (Figure 4C) (Supplementary Figure S4C). By quantitatively analyzing and comparing the number and volume of tumor masses formed at 20 weeks, LPL, FABP4 or CPT1 inhibitors significantly reduced liver carcinogenesis in STAM mice, especially in the Orlistat group and BMS309403 group (Figure 4D, E).

### Effects of LPL/FABP4/CPT1 molecular activity on the formation of liver cancer stem cells

**Figure 5:**
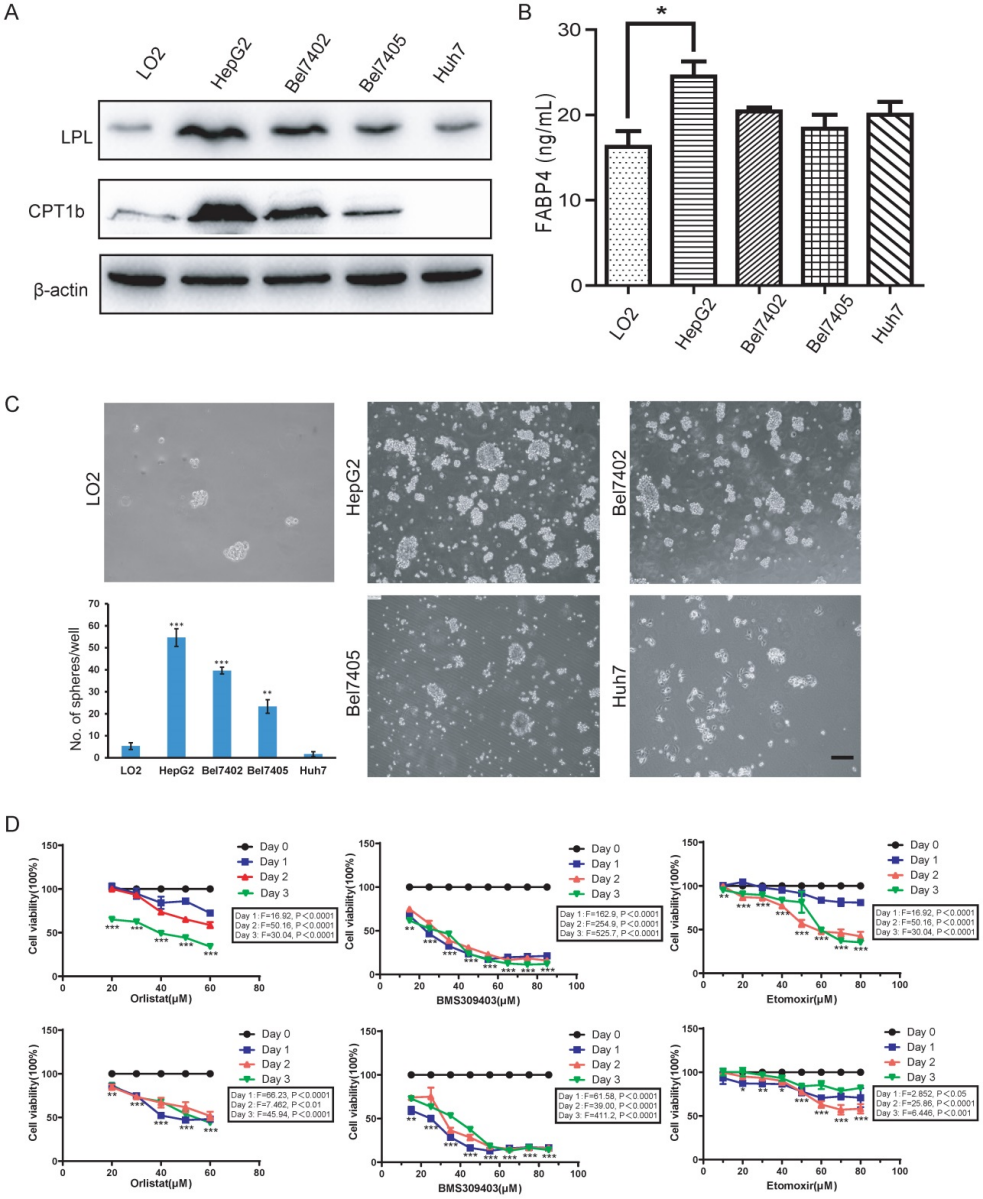
** The LPL/FABP4/CPT1 molecules regulate the ability of spheroid formation of HCC cell lines. A,** Western blot analysis of LPL and CPT1b in human normal hepatocytes LO2, and other different HCC cell lines. β-Actin was used as a loading control. **B,** By ELISA analysis, FABP4 was found to be highly expressed in the supernatant of HCC cell lines, especially in HepG2 (*P < 0.05 vs Control (LO2), n = 3 independent experiments). **C,** LO2, HepG2, Bel7402, Bel7405 and Huh7 cells were used for spheroid formation assay. A paired two-tailed Student's t-test was used for statistical analysis. Error bars = mean ± S.D (Scale bars, 100 μm, n=4 independent experiments). **D,** MTT results showed that the inhibition of LPL/FABP4/CPT1 molecular activity can slow down the proliferation of HepG2 and Bel7402 cells (n = 3 independent experiments). The difference of inhibition levels between different concentrations at the same time point (data in the box) and the difference of inhibition levels between the same concentration at different time points (represented by *, **, ***) were obtained by one-way ANOVA analysis.

Since fatty acid oxidation is important for the self-renewal of tumor-initiating cells 26, the activation of the fatty acid metabolic signal axis during NASH progression may create a tissue microenvironment conducive to tumorigenesis by promoting the activity of LCSCs. To test this hypothesis, we used the method of tumor spheroid culture *in vitro* to enrich the LCSCs in different HCC cell lines. Then, the biological behavior of the LCSCs treated with LPL, FABP4 and CPT1 inhibitors (i.e., Orlistat, BMS309403 and Etomoxir) were evaluated. Western blot in the normal liver cell line LO2 and four HCC cell lines (HepG2, Bel7402, Bel7405 and Huh7) showed that the expression of LPL and CPT1b protein was higher in HepG2 and Bel7402 cell lines (Figure 5A). Given that FABP4 is a secretory protein, the ELISA assay was applied to detect the content of FABP4 in the supernatant of the culture medium, and it was found that the content of FABP4 in the supernatant of HepG2 cell culture medium was higher than in those of the other cells (Figure 5B). The tumor spheroid formation experiment showed that, compared with the LO2 cell line, the number of spheroid cells formed in HepG2 and Bel7402 cells was significantly bigger, indicating that HepG2 and Bel7402 had the stronger spheroid-forming ability (Figure 5C). Bel7405 had a smaller number of spheres, while Huh7 could hardly form spheres. Corresponding to western blot results (Figure 5A), the higher the expression level of the LPL/FABP4/CPT1 molecules, the stronger the spheroid-forming ability of LCSCs. These data suggested that activation of the LPL/FABP4/CPT1 axis is conducive to the enhancement of LCSCs activity. Based on the above results, HepG2 and Bel7402 HCC cells were selected for MTT analysis to study the effects of inhibitors of these molecules on HCC cell proliferation. The results showed that the cell activity of HepG2 and Bel7402 decreased gradually with the increasing concentration of inhibitor drugs (Figure 5D), indicating that inhibition of the LPL/FABP4/CPT1 axis could retard the proliferation of HCC cells. Taken together, these results demonstrated higher activity of the LPL/FABP4/CPT1 axis is positively correlated with the formation and proliferation of LCSCs.

**Figure 6:**
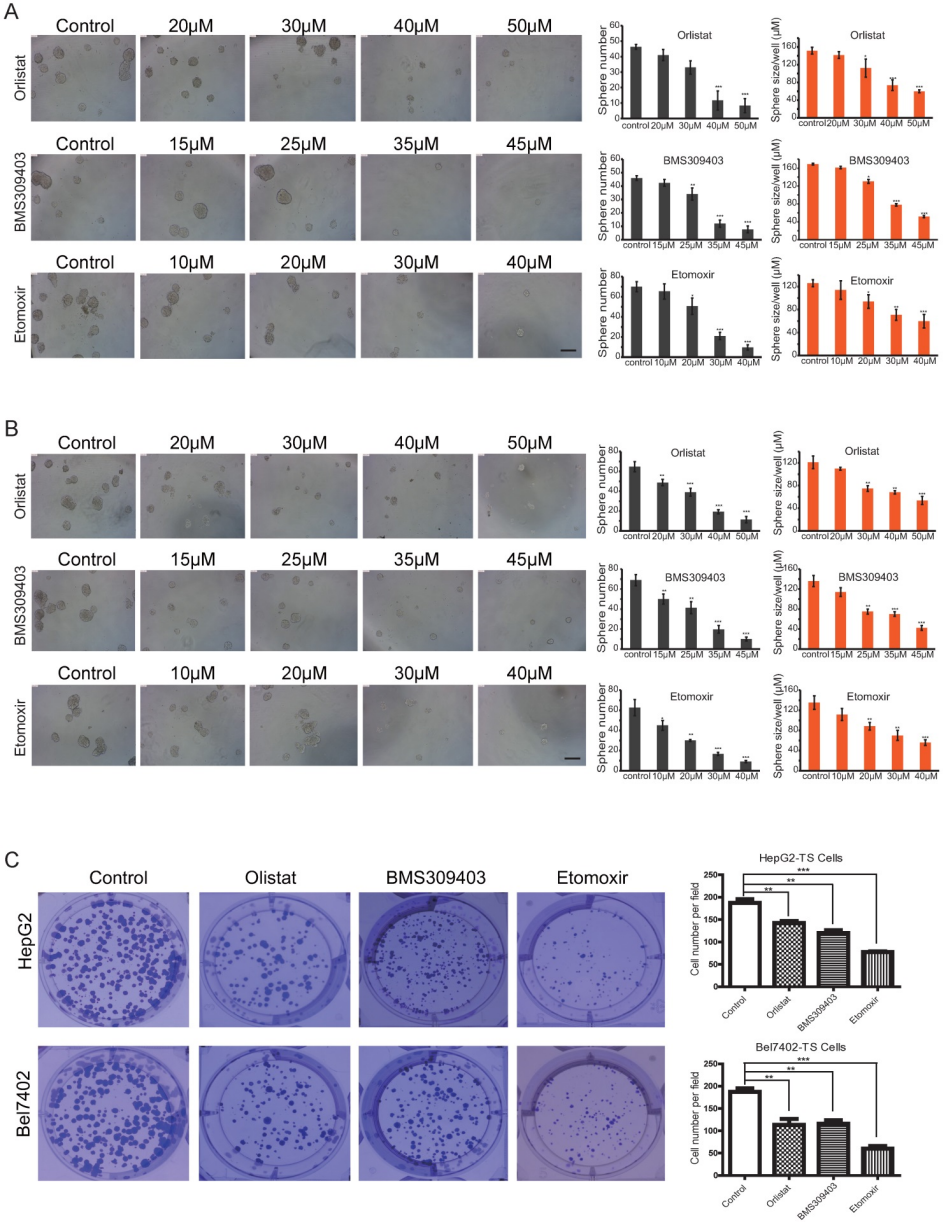
** LPL/FABP4/CPT1 molecular inhibitors inhibit the self-renewal of LCSCs.** Tumor spheres (TS) were collected for the second round of spheroid formation assay. HepG2-TS **(A)** and Bel7402-TS **(B)** cells were again subjected to a spheroid formation assay supplied with LPL, FABP4 and CPT1 inhibitors. The size and volume of the spheroids of these cells were significantly decreased after the treatment of inhibitors. **C,** Under the administrations of LPL, FABP4 and CPT1 molecular inhibitors, HepG2-TS and Bel7402-TS cells were subjected to plate colony formation experiments. Representative pictures for colony growth are shown (10 cm dishes). Quantification of the number of colonies was shown. A paired two-tailed Student's t-test was used for statistical analysis. Data are shown as mean ± S.D. (*P < 0.05, **P < 0.01, ***P < 0.001 vs Control, n = 3 independent experiments).

To analyze the effect of different concentrations of inhibitors on the self-renewal abilities of LCSCs, microsphere formation assays were conducted by using Passage 3 (P3) LCSCs of HepG2 and Bel7402 cells (HepG2-TS and Bel7402-TS). As shown in Figures 6A and B, all three inhibitors could inhibit the self-renewal of LCSCs in a dose-dependent manner. A direct comparison of the effects of these three inhibitors indicated that Orlistat showed the strongest inhibitory effect on LCSCs at 30 μM, BMS309403 had the best inhibitory effect at the concentration of 35 μM, and Etomoxir had the best inhibitory effect at 30 μM (P<0.001). Consistent results were obtained by genetic interference with Crispr/cas9 system targeting LPL or CPT1b genes (Supplementary Figure S5A, B). To test whether three inhibitors of LPL/FABP4/CPT1 molecules affect the clonogenicity of HCC stem cells, we carried out an *in vitro* clonal formation experiment. The results showed that all three inhibitors could effectively inhibit the clone formation of LCSCs *in vitro*. The size and number of two types of LCSCs clones were dramatically reduced upon administration of inhibitors. The above results suggest that Inhibition of LPL/FABP4/CPT1 molecular activity may be an effective intervention method for LCSCs proliferation and self-renewal (Figure 6C).

## Discussion

NAFLD/NASH represents an increasingly important risk factor for liver carcinogenesis. In recent years, dramatic changes in diet and lifestyle have contributed to the growing prevalence of obesity and NAFLD worldwide. The proportion of NAFLD/NASH-associated liver cancer might increase further. However, the key molecular events underlying the progression of NASH to HCC are poorly understood, leading to a lack of interventions. In this study, we first analyzed the gene expression profile of STAM mice at different stages of NAFLD progression and observed that the activation of the LPL/FABP4/CPT1 molecular axis that controls lipid metabolism may be involved in the progression of NASH to HCC. GSVA analysis indicated a widespread upregulation of a variety of oncogenic signals occurs upon NASH, which may drive the hepatocyte-LCSCs transdifferentiation. We also found that with the aggravation of NAFLD, the expression of LPL, FABP4 and CPT1 molecules in liver tissues also increased. Importantly, *in vivo* tests showed that LPL, FABP4, and CPT1-related inhibitors delayed tumor growth in STAM mice. *In vitro* experiments also confirmed that the inhibition of these signaling molecules could effectively reduce the tumor spheroid formation, indicating that the activation of the LPL/FABP4/CPT1 axis in the NASH stage may be beneficial to the viability of TICs in liver tissue.

The LPL/FABP4/CPT1 axis, which controls fatty acid metabolism, has multiple biological functions and is the focus of this study. LPL is a rate-limiting enzyme that promotes the decomposition of TG-rich circulating lipoproteins, including CM, LDL and VLDL 29, 30. Some studies have raised the possibility that glioma cells rely on LPL molecules for fuel and that glioma cells have higher levels of free fatty acids than normal brain tissue 31. FABP4 is a lipid-accompanying protein of the 14-15 kDa family, which has a high affinity for hydrophobic ligands (including saturated and unsaturated long-chain fatty acids) 32. FABP4 has been shown to play a key role in insulin resistance, type 2 diabetes and atherosclerosis 33, 34. Besides, the level of FABP4 in the systemic circulation is up-regulated in patients with NAFLD, which is associated with liver inflammation and fibrosis 35. Fat cells around malignant cells use FABP4 to provide FA to malignant cells to maintain cell proliferation 36. Recent data reported that FABP4 was increased in liver tissue of HCC models that were chemically induced with a high-fat diet (HFD) 37. The specific endothelial overexpression of FABP4 in MS-related human HCC and its carcinogenic effect has been verified 38. CPT1 is the major factor regulating FA oxidation (FAO) and maintaining energy homeostasis 39. Because CPT1 promotes the entry of fatty acids into the mitochondrial matrix, which plays a vital role in β-oxidation to produce energy, CPT1 has become a potential therapeutic target in cancer. Several studies have shown that Etomoxir can induce anti-proliferation by inhibiting CPT1a and CPT1b 40. Other studies have shown that low expression of CPT1b inhibits the survival of breast cancer initiation cells and knockdown of CPT1c expression effectively reduces breast cancer xenografts 25, 41. Conclusively, three key genes, LPL, FABP4 and CPT1, form the metabolic axis that regulates fatty acid production, transport and oxidization, which is essential for cancer cell activity. The activation of this signal axis in NASH and HCC samples may affect tumorigenesis by improving tumor-initiating cell viability.

The STAM mice provide the advantage of monitoring the natural progression of NAFLD in a relatively controlled manner. The expression changes of the LPL/FABP4/CPT1 axis with time during the evolution from NASH to HCC in STAM mice were verified. From 8-week to 20-week of the STAM mice model, we used small-molecule inhibitors targeting LPL, FABP4 or CPT1 for intervention. By reviewing the literature, the concentrations of small-molecule inhibitors Orlistat, BMS309403 and Etomoxir were determined to be 100 mg/kg 42 , 15 mg/kg 43, 44 and 15 mg/kg 45, 46. We observed that the intraperitoneal injection of small-molecule inhibitors could effectively reduce the number and size of liver tumors in STAM mice, indicating that NASH-related tumorigenesis and tumor growth were inhibited. In particular, Orlistat is most obvious, possibly because LPL is at the beginning of the process of breaking down chylous droplets absorbed by the liver, providing fatty acid for the proliferation of liver cancer cells. Because Orlistat is an FDA-approved drug for weight loss, its role in inhibiting NASH-related HCC would be of clinical significance.

The development and progression of HCC are linked with the existence of LCSCs. The existence of CSC niche and plasticity behavior makes CSC quickly change from static to proliferative, follow asymmetric division, and change from initial cell to non-initial cell state, which may be the reason for drug resistance and recurrence in cancer treatment. CSCs have been identified in a variety of cancers 47, 48. HCC is heterogeneous, containing cells with initial cell-like characteristics located at the top of the cell hierarchy 49. In this study, the results of tumor spheroid culture and colony formation *in vitro* showed that small-molecule inhibitors of the LPL/FABP4/CPT1 axis, including Orlistat, BMS309403 and Etomoxir, could inhibit the activity of LCSCs. The activation of fatty acid metabolism regulated by the LPL/FABP4/CPT1 axis may provide a tumor microenvironment conducive to the production of LCSCs, although the detailed mechanism remains to be further explored.

In summary, after analyzing the gene expression data, we found that the LPL/ FABP4/CPT1 molecular axis was up-regulated along with the cell proliferation genes in the progression of NAFLD/NASH to HCC. The LPL/FABP4/CPT1 axis can control lipid decomposition, fatty acid supply and fatty acid oxidation processes in the liver. Since the progression of liver cancer depends on oxidative phosphorylation and fatty acid metabolism to facilitate the self-renewal and tumor-initiation abilities of LCSCs, Activation of the LPL/FABP4/CPT1 axis in NASH may represent a metabolic reprogramming that promotes tumorigenesis by nurturing oncogenic signal-driven TICs, thus promoting the development of NASH to HCC. Of particular importance, we demonstrate that inhibition of this process can prevent NAFLD/NASH from developing into HCC. Therefore, it may provide a potentially effective method for the prevention of NASH-related HCC by targeted inhibition of the LPL/FABP4/CPT1 axis. Further studies are needed to clarify in detail the exact molecular mechanisms and the *in vivo* efficacy and safety of this intervention strategy.

## Materials and Methods

### Microarray data mining and gene expression analysis

The STAM mouse data set used in this study comes from the GEO database (GSE83596). The chip data was submitted by De Conti A et al. on June 21, 2016. The RNA-seq data of the Liver Hepatocellular Carcinoma (LIHC) samples was downloaded from the TCGA database. The R package “limma” was applied to process the gene expression matrix. Differentially expressed genes (|log_2_(FC)| > 1, P < 0.05) shared in different samples were analyzed by a Venn diagram. The microarray data of liver biopsy tissues of human obese patients was used for verification of DEGs, which was downloaded from the GEO database (GSE66676) provided by Xanthakos S on March 9, 2015.

### Functional enrichment analysis, PPI network construction and GSVA analysis

Shared DEGs were imported into the STRING (Version 10.0, https://www.string-db.org/) web tool to map the protein-protein interaction (PPI) network. The plugin MCODE in Cytoscape software (http://www.cytoscape.org/) was used to analyze the PPI network for functional modules of DEGs. The DAVID tool (the Database for Annotation, Visualization and Integrated Discovery) or R package clusterProfiler was applied for KEGG pathway analysis and visualization (P <0.05). The GSVA was applied to explore the differences in oncogenic signature between normal and STAM mice using the oncogenic signature gene sets (https://www.gsea-msigdb.org/gsea/msigdb) and analyzed by the R package “GSVA”.

### Feed, drugs and antibodies

High-fat feed diet (fat calories 60%) and basic control feed diet (fat calories 7%) are SPF-grade feeds, provided by Trophic Animal Feed High-tech Co., Ltd (China). Streptozocin (STZ, S0130), LPL inhibitor Orlistat (HY-B0218), FABP4 inhibitor BMS309403 (HY-101903) and CPT1 inhibitor Etomoxir (HY-50202), all were purchased from MedChemExpress (MCE). LPL and CPT1b mouse monoclonal antibodies were purchased from Boster Biotechnology Co., Ltd. FABP4 polyclonal antibody was purchased from Cell Signaling Technology Company. EpCAM polyclonal antibody was purchased from R&D Systems. The rabbit anti-actin antibody was ordered from Fuzhou Maixin Biotechnology Company (China) or the company of Shenggong Biotechnology Co., Ltd. (China), respectively.

### Animal model preparation

60 C57BL/6 mice including 20 males and 40 females, (SPF grade, 7-8 weeks old, weighs 18-22 grams), were supplied by the Experimental Animal Center of Anhui Medical University. Male and female mice will be caged to breed offspring, and male pups will be randomly divided into the following groups: Normal group, STAM model group. The mice in the Normal group were fed a normal diet and drinking water for 20 weeks; the STAM model group was established as follows: 2-day-old male mice were subcutaneously injected with streptozotocin (STZ, 200 μg/mice). Then the male mice were then fed a high-fat diet to construct the STAM mice model after 4 weeks of age. Inhibitor intervention was initially given at 8-week age. I.e., male mice in the S+O group, S+B group and S+E group were intraperitoneally injected with Orlistat (100 mg/kg) and BMS309403 (15 mg/kg) and Etomoxir (15 mg/kg) at 8 weeks for every two days. The inhibitor solutions were formulated according to the same protocol. Briefly, each inhibitor was dissolved by adding the following solvent in turn: 10% DMSO → 40% PEG300 → 5% Tween-80 → 45% saline. The STAM control group was injected intraperitoneally with saline containing the above solvent mixture as control. During the intervention, the mice were monitored for diet, fur color and death, and weighed once a week. Liver index = liver weight/body weight×100%, tumor volume V (mm^3^) = L × B^2^/2 (L: tumor length in mm; B: tumor width in mm). All protocols and procedures are approved by the Ethics Committee of Animal Experiments at the Hefei Institutes of Physical Science, Chinese Academy of Sciences. All animal experiments comply with the ARRIVE guidelines and were carried out following the National Institutes of Health guide for the care and use of laboratory animals (NIH Publications No. 8023, revised 1978).

### Biochemical examination

The blood samples of mice were collected after 6 hours of fasting. Then left for 30 minutes to centrifuge at 3000 r/min for 10 minutes, and the serum was collected for analysis. Alanine aminotransferase (ALT), aspartate aminotransferase (AST), total cholesterol (TCH), triglyceride (TG), high-density lipoprotein (HDL) and low-density lipoprotein (LDL) levels in mice were measured using UniCel Dxl800 (Beckman Coulter).

### Immunohistochemistry

Paraffin-embedded, formalin-fixed tissue sections were dewaxed in a gradient solution of xylene and then rehydrated in 100% ethanol and 1× PBS buffer. It was then boiled in 0.1 mol/L citric acid buffer (pH 6.0) for 10 minutes for antigen repairing and subsequently sealed with 10% goat serum at 37 °C for 60 minutes. The tissues in slides were incubated overnight with the primary antibody at 4 °C, then washed with 1× PBS buffer and incubated with the secondary antibody for 30 minutes, and then stained with DAB (3, 3'-Diaminobenzidine). The nuclei were counterstained with hematoxylin for 2 minutes. IPP 6.0 (Image Pro Plus 6.0) was used to measure the protein expression of regional mean optical density (MOD) value for statistical analysis.

### Plasmid Construction, Lentivirus Packaging, and Infection

The Cas9 stable-expressing cells were established by lentiviral transduction of Cas9 coding sequence into the genome of cells. The sgRNAs sequences targeting LPL and CPT1b were designed by CRISPOR Design web tool: https://zlab.bio/guide-design-resources. All the sgLPL and sgCPT1b sequences are listed as follows: LPL-KO-1-sg-F, CACCGGTCTGACCGCCTCCCGCGGA; LPL-KO-1-sgDNA-R, AAACTCCGCGGGAGGCGGTCAGACC; LPL-KO-2-sgDNA-F, CACCGGTCCGCGGGCTACACCAAAC; LPL-KO-2-sgDNA-R, AAACGTTTGGTGTAGCCCGCGGACC; CPT1b-KO-sgDNA-F, CACCGGATCATGTATCGCCGTAAAC; CPT1b-KO-sgDNA-R, AAACGTTTACGGCGATACATGATCC. For establishing a stable knockdown cell model, plasmid vectors including lentiCRISPRv2, psPAX2 and pMD2.G were used for lentivirus packaging and then infection of cells. The effect of gene knockout was verified by western blot analysis.

### Western blot

Cultured cells or frozen tissues were lysed in Laemmli buffer containing a proteinase inhibitor cocktail (Santa Cruz). After centrifugation, the supernatant was harvested, and the protein concentration was detected by the BCA protein detection kit (Pierce). For immunoblotting, 15-30 µg of protein was separated by SDS-PAGE and then transferred to the PVDF membrane. The membrane was then blocked with 5 % bovine serum albumin in Tris-buffered saline containing Tween-20 (TBST). The membrane was incubated with the primary antibody overnight at 4 °C, then washing the membrane with TBST before incubation with the secondary antibody. Target proteins were detected with corresponding 1st and 2nd antibodies and visualized using Western bright ECL HRP substrate (Advansta Inc.). The primary antibody against β-actin was used as a control, and the result was defined by the ratio of target protein/β-actin.

### ELISA assay

An ELISA Kit (Kete Biological Technology Co., Ltd, Jiangsu, China) was used to measure the level of FABP4 in the cell culture medium. The experiment was carried out according to the instruction manual provided by the company. Briefly, adding the collected cell culture fluid and standard products to the bottom of the ELISA plate, sealing the plate, and incubating at 37 °C for 30 min. After incubation, the reaction mixture was discarded and the plate was washed five times with a cleaning solution. After washing, successively mix 50 μl of developer A and B into the wells, mix well for 15 min, and then add 50 μl of stop solution to terminate the reaction. Within 15 min after terminating the reaction, using the blank hole to adjust the zero, and the SpectraMax absorbance reader (Molecular Instruments) was applied to read the absorbance (OD value) at 450 nm.

### MTT assay

The HepG2 and Bel7402 cell lines in the logarithmic growth phase were seeded in a 96-well plate at a density of 5×10^3^ cells/well. After 24 h of culture, the serum-free medium was replaced for 12 h, and different concentrations of inhibitors were added to the cell culture medium of each treatment group in the 96-well plate. Cells were incubated at 37 ℃ for 72 h. The chromogenic time for cells was at 0, 1st, 2nd and 3rd day, respectively. In the dark, MTT (5 mg/mL) was added to the 96-well plate with a volume of 20 μl/well, and the culture was further incubated for 4 h. Then, the medium was discarded, and the DMSO solution of 150 μl/well was added, then plates were shaken at a low speed for 15 min. After the violet crystal is completely dissolved, the absorbance (OD) is read at 570 nm using the SpectraMax absorbance (molecular instrument).

### Cell lines

Human cell lines used in this study include normal liver cells LO2 and liver cancer HepG2, Bel7402, Bel7405, Huh7, purchased from American Type Cell Culture (ATCC), and cultured in recommended media at 37 °C, 5% CO2 in a Thermo Fisher X50 incubator.

### Stem cell suspension and LCSCs enrichment

The LO2, HepG2, Bel7402, Bel7405, and Huh7 cell lines were cultured using a serum-free suspension method to form tumor spheres (TS). When the cell lines reached 80% confluence, the medium will be replaced with a fresh one. The cells were digested with trypsin, and then a complete culture medium containing 10% fetal bovine serum was added and thoroughly mixed to form a cell suspension. After centrifugation, the supernatant was discarded and the cells were inoculated in a 6-well plate with ultra-low adhesion at 1×10^5^ cells/ml. DMEM/F12 medium was utilized to culture cells for about 5-7 days. When the size of single-cell spherules reached 60-100 μm, the larger ones were collected at low-speed centrifugation. The cells were re-trypsinized to form a single-cell suspension, and then cultured again in DMEM/F12 medium for 5-7 days. This procedure was repeated until the third-generation (P3) of LCSCs was obtained. The P3 LCSCs were harvested for subsequent assays.

### LCSCs microsphere formation assay

HepG2 and Bel7402 cells were cultured in a serum-free medium for three generations and tumor spheroid cells (HepG2-TS and Bel7402-TS) were obtained for follow-up LCSCs microsphere formation and immunofluorescence assays. For microsphere formation assay, the tumor spheroid cells were digested into a single-cell suspension. Then each well was inoculated with 100 μl at a density of 4×10^3^ cells/mL in a 96-well plate. Different concentrations of inhibitors were added and the status of microsphere formation was examined every day.

### Immunofluorescence

Cells were fixed in 4% cold paraformaldehyde and then permeabilized by 0.2% Triton X-100 for 30 minutes. The slides were blocked with 5% bovine serum albumin buffer for 1 hour at 37 °C and were then incubated with primary antibodies at room temperature for 1-2 h or 4 °C overnight, followed by cultivating with secondary antibodies for 1 hour. Later, cells were rinsed and counterstained using DAPI (Sigma).

### Plate colony formation assay of cancer stem cells

P3 generation spheroid cells (HepG2-TS and Bel7402-TS) were applied to plate colony formation assay. Cells at a density of 5×10^5^ cells/mL were inoculated in a 6-well plate, placing them in an incubator for 24 h. Cells were then digested and cell density was adjusted with gradient dilution in a 6-well plate. Inhibitors with the optimal concentration were added and cells were placed in an incubator for 2-3 weeks until a single clone is formed. The cell colonies were stained with crystal violet.

### Statistical Analysis

Comparisons of data were performed with a paired two-tailed Student's t-test or one-way ANOVA test. Data are presented as means ± SD. When the P-value was less than 0.05, the difference was considered statistically significant.

## Supplementary Material

Supplementary figures.
