# Supplementary material for: Targeted Inhibition of LPL/FABP4/CPT1 fatty acid metabolic axis can effectively prevent the progression of nonalcoholic steatohepatitis to liver cancer
Source: Int J Biol Sci. 2021 Oct 11;17(15):4207–22. doi: 10.7150/ijbs.64714 (PMC8579444; doi:10.7150/ijbs.64714)
Supplement: Supplementary file 1 — Supplementary figures. [file ijbsv17p4207s1.pdf]

Supplementary figure legends

Supplementary figure S1

Common DEGs are enriched in the pathway of "Cell cycle". A, Kyoto Encyclopedia of Genes and Genomes (KEGG) pathway analysis shows the most enriched pathway for common DEGs is related to the process of cell cycle. B, The protein-protein interaction (PPI) network of common DEGs was constructed by using the STRING database.

Supplementary figure S2

Blood lipid-related index analysis of STAM mice. (*P < 0.05, **P < 0.01, ***P < 0.001, n=5 in each group).

Supplementary figure S3

The localization of LPL/FABP4/CPT1 molecules. A, IHC imaging to show the staining of LPL, FABP4 and CPT1b in liver tissues of STAM mice. (scale bars, 100 μm). B, Co-immunostaining of CPT1b with EpCAM marker (scale bars, 50 μm).

Supplementary figure S4

Comparison of the physiological indexes between inhibitors-treated and normal STAM mice. Liver weight ratio (A) and serum-related indexes (B) are compared between the STAM group and the other three inhibitor-treated groups. C, The representative images of HE staining showed the histological features of the liver tissues for STAM mice at 20 weeks, compared with those of normal mice (scale bars, 100 μm). (*P < 0.05, **P < 0.01, ***P < 0.001, n=5 in each group).

Supplementary figure S5

The knockdown of LPL and CPT1b decreases the ability of spheroid formation of HCC cell lines. A, Western blot analysis of LPL and CPT1b knockdown cells. β-Actin was used as a loading control. B, LPL deficient cells were used for spheroid formation

assay. C, CPT1b deficient cells were used for spheroid formation assay.

A

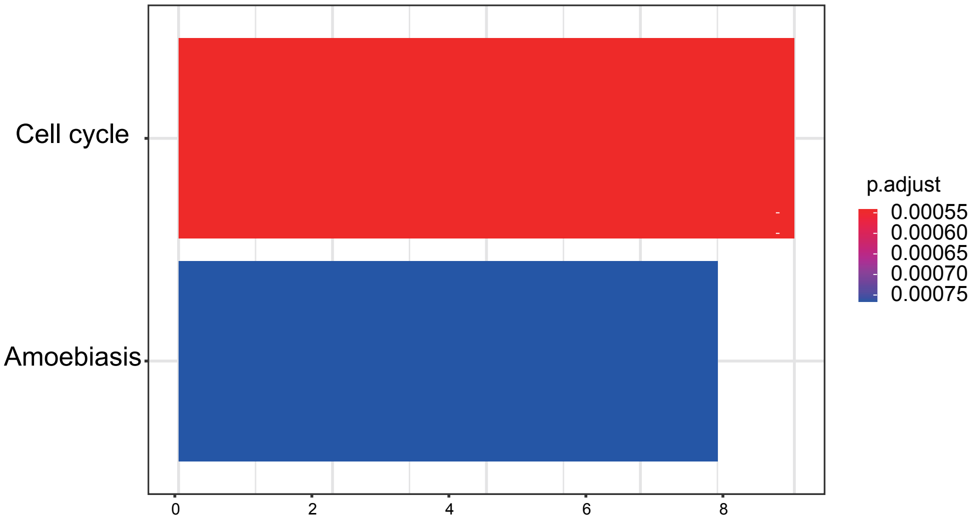

B

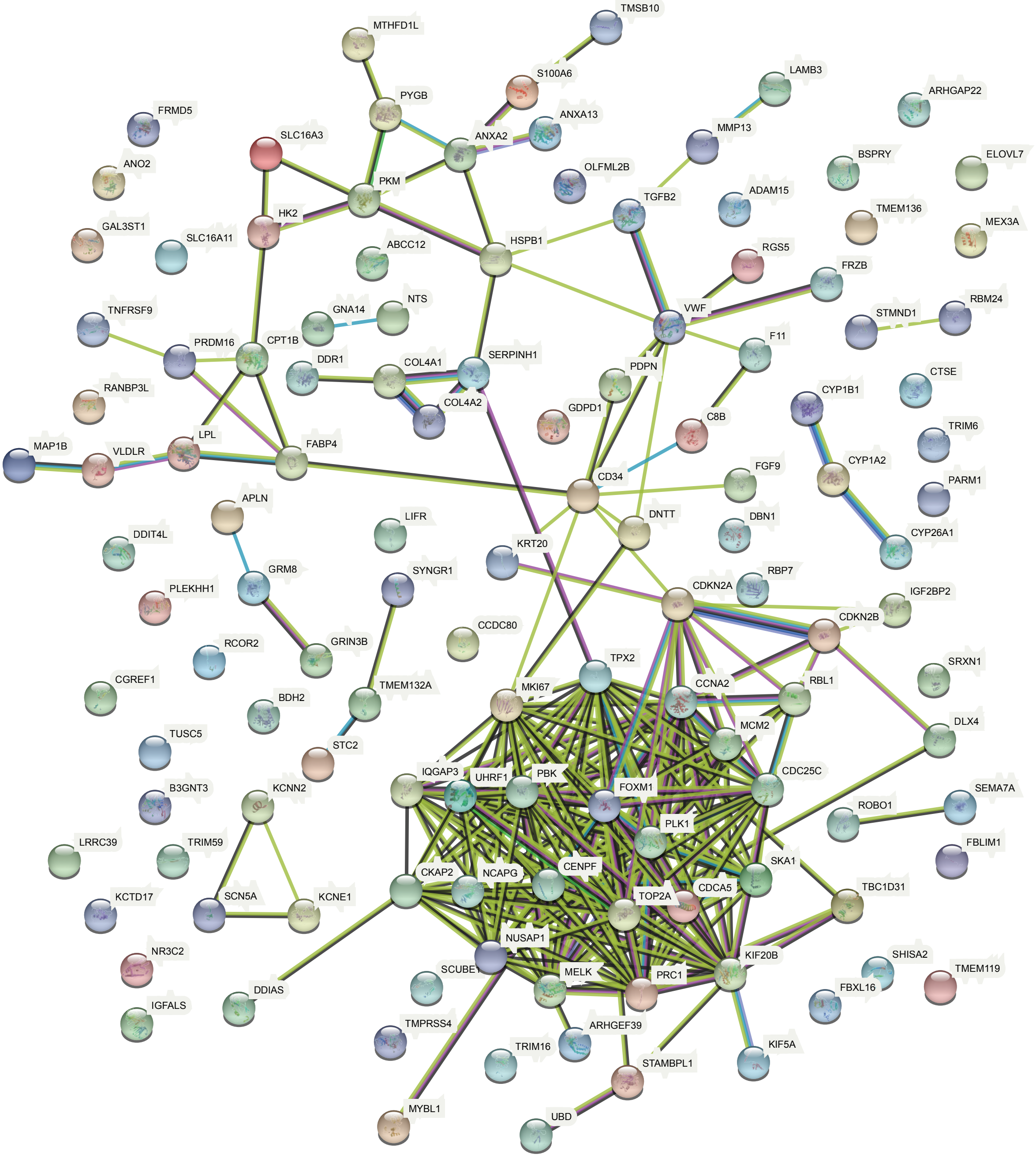

Supplementary Figure S1

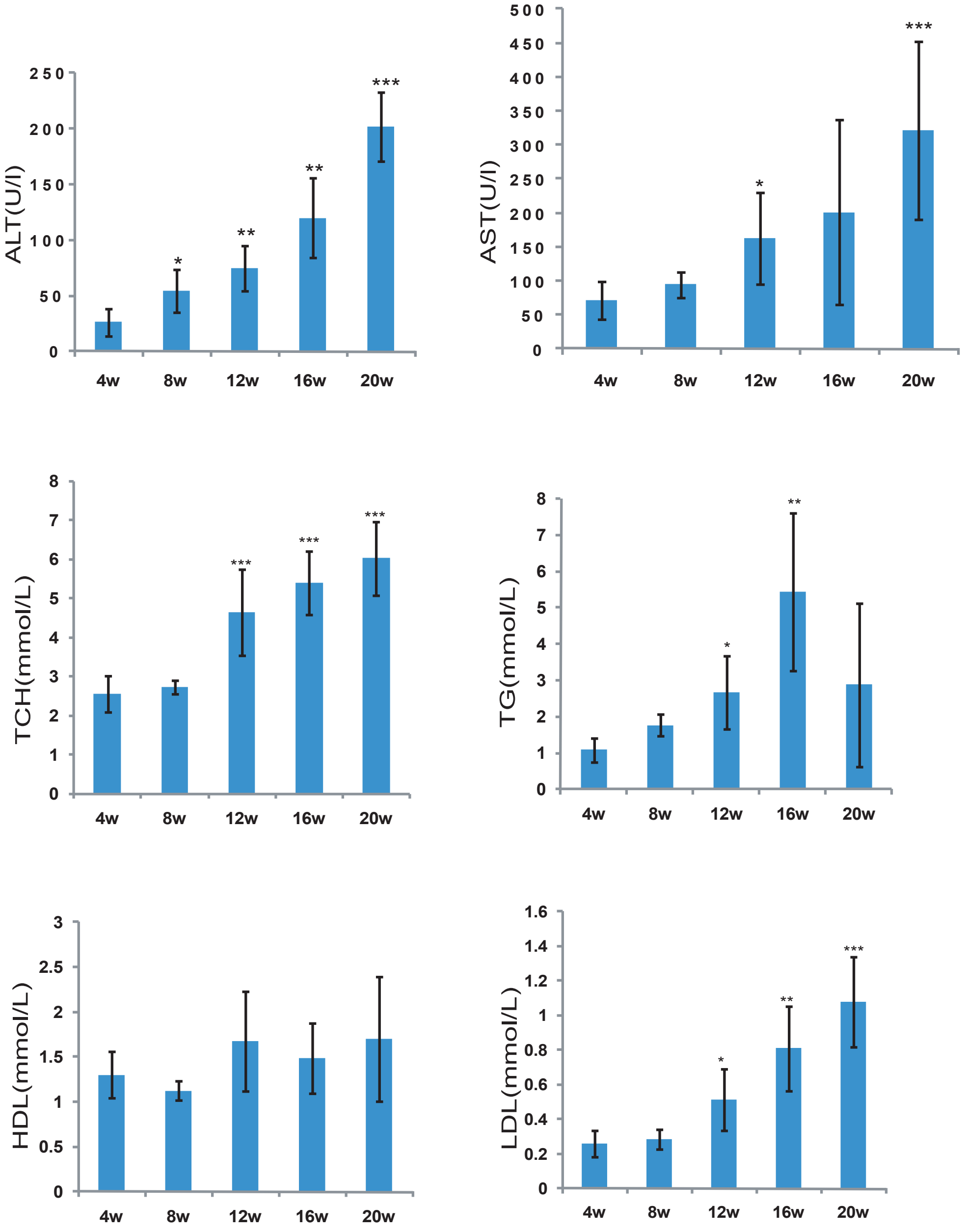

Supplementary Figure S2

A

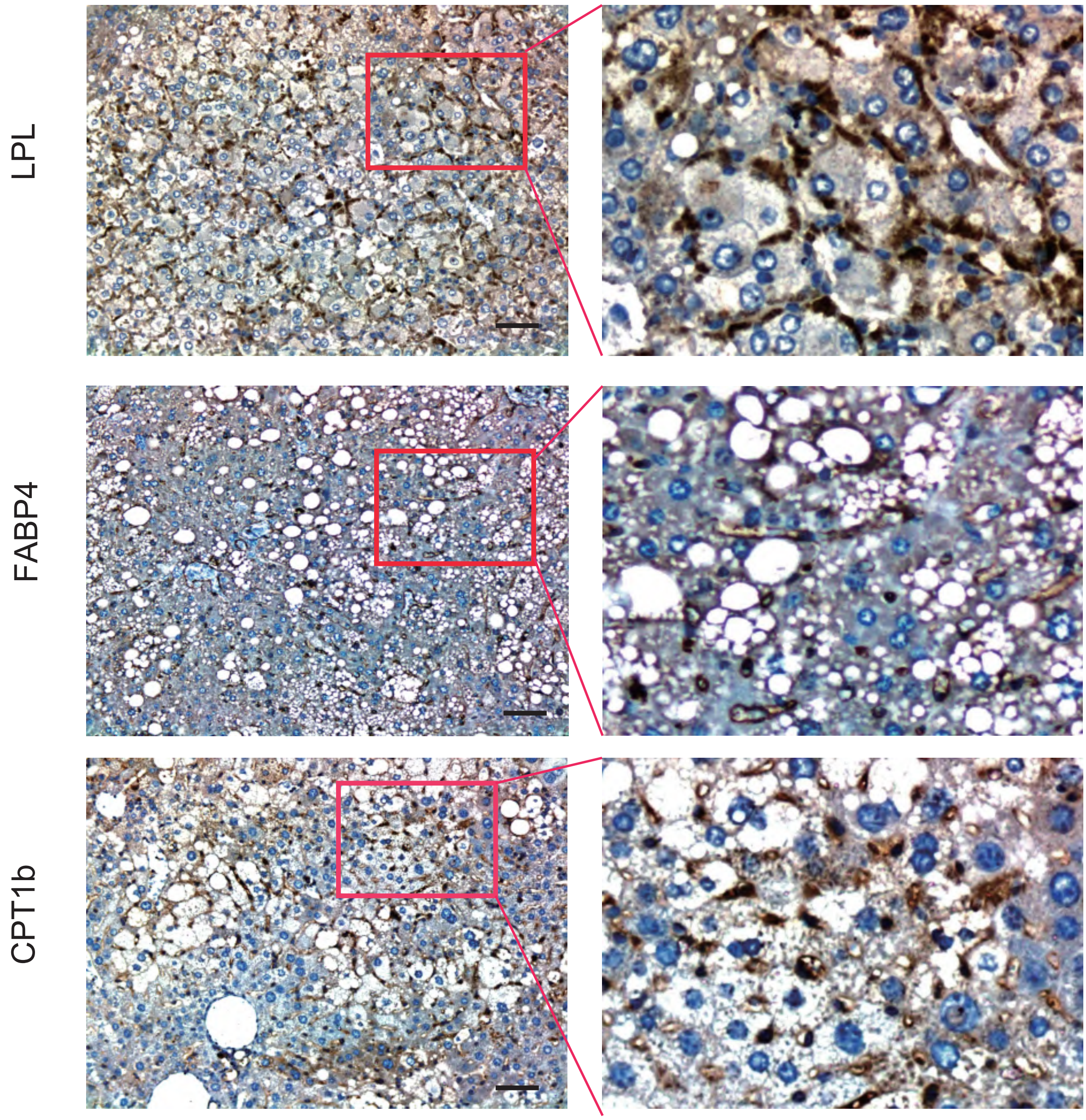

B

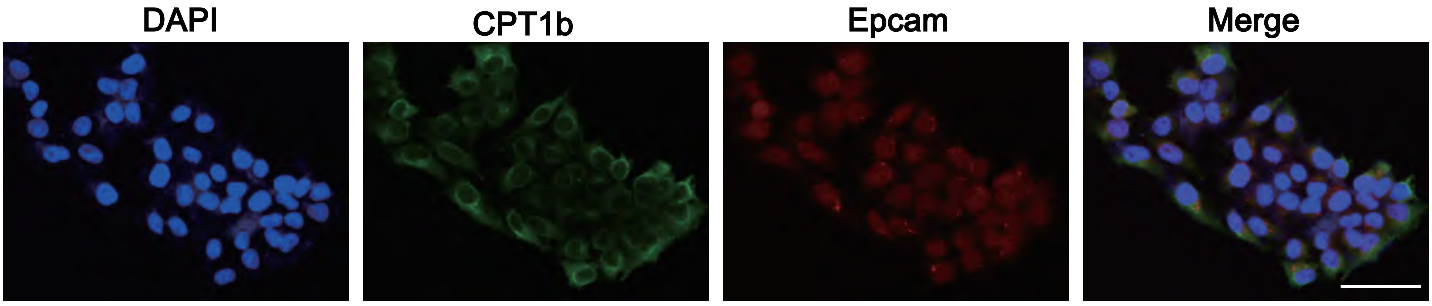

Supplementary Figure S3

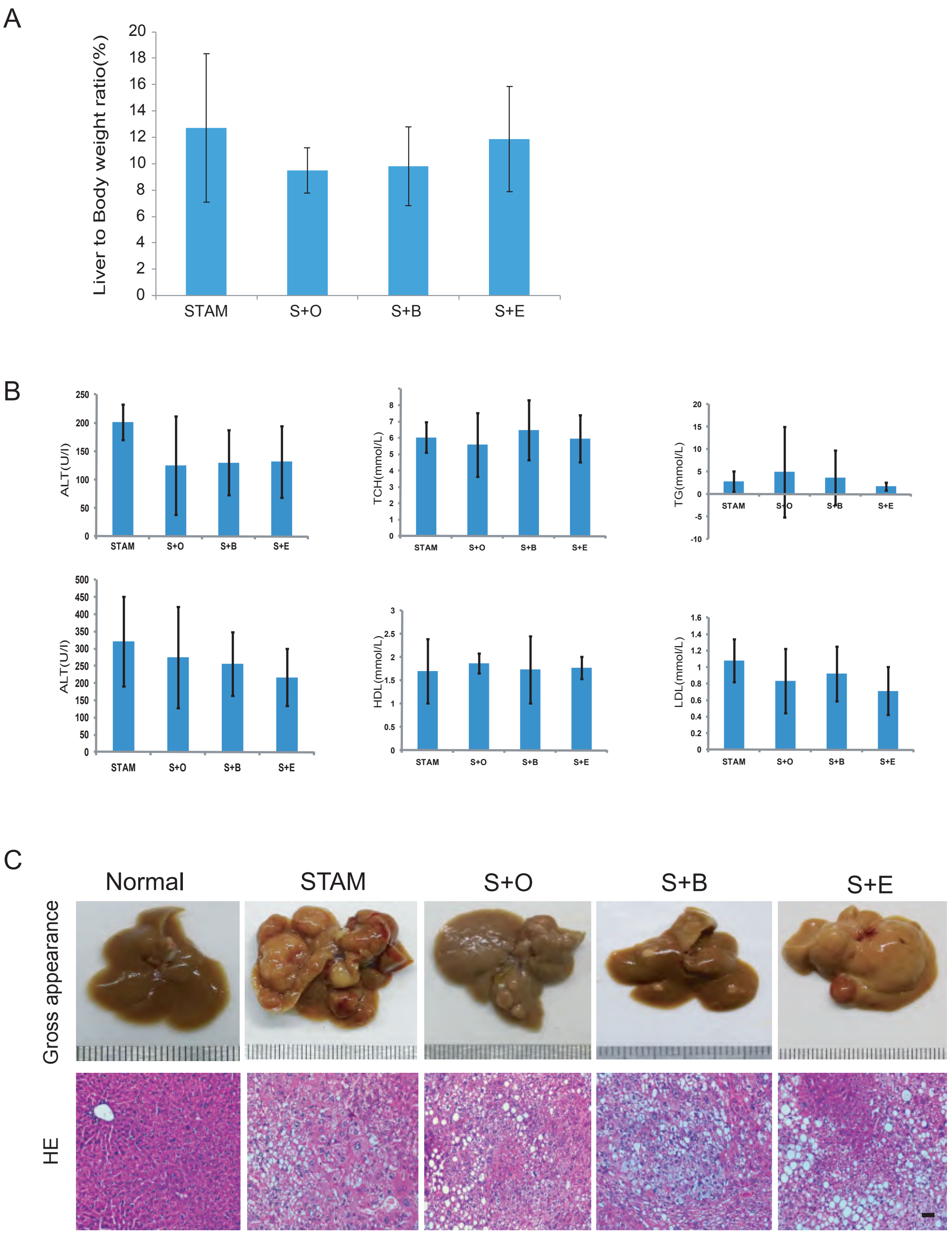

Supplementary Figure S4

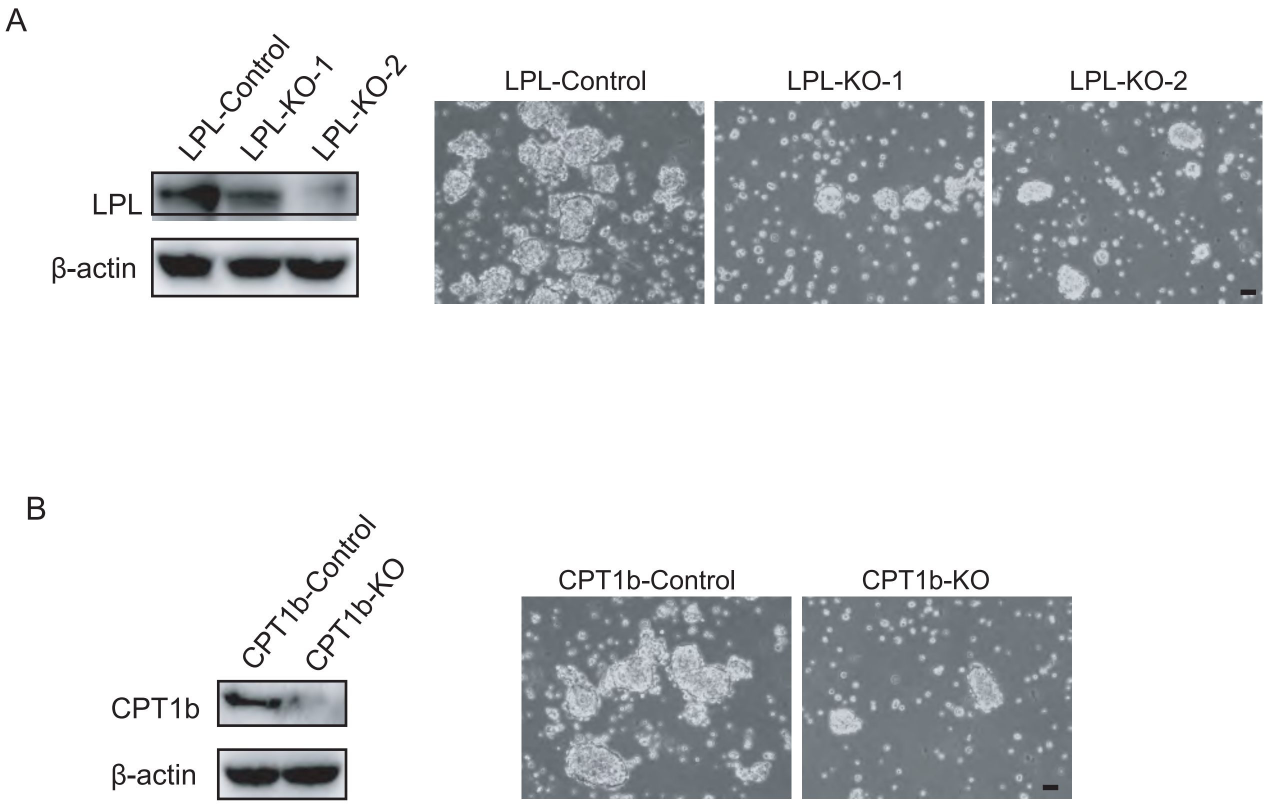

Supplementary Figure S5
